# Metabolomics analysis and metabolite‐agronomic trait associations using kernels of wheat (*Triticum aestivum*) recombinant inbred lines

**DOI:** 10.1111/tpj.14727

**Published:** 2020-03-31

**Authors:** Taotao Shi, Anting Zhu, Jingqi Jia, Xin Hu, Jie Chen, Wei Liu, Xifeng Ren, Dongfa Sun, Alisdair R. Fernie, Fa Cui, Wei Chen

**Affiliations:** ^1^ National Key Laboratory of Crop Genetic Improvement and National Center of Plant Gene Research (Wuhan) Huazhong Agricultural University Wuhan 430070 China; ^2^ College of Plant Science and Technology Huazhong Agricultural University Wuhan 430070 China; ^3^ Max‐Planck‐Institute of Molecular Plant Physiology Potsdam‐Golm 14476 Germany; ^4^ Wheat Molecular Breeding Innovation Research Group Key Laboratory of Molecular Module‐Based Breeding of High Yield and Abiotic Resistant Plants in Universities of Shandong School of Agriculture Ludong University Yantai China

**Keywords:** *Triticum aestivum* L., mature seed, metabolic quantitative trait loci, agronomic trait, metabolic prediction

## Abstract

Plants produce numerous metabolites that are important for their development and growth. However, the genetic architecture of the wheat metabolome has not been well studied. Here, utilizing a high‐density genetic map, we conducted a comprehensive metabolome study via widely targeted LC‐MS/MS to analyze the wheat kernel metabolism. We further combined agronomic traits and dissected the genetic relationship between metabolites and agronomic traits. In total, 1260 metabolic features were detected. Using linkage analysis, 1005 metabolic quantitative trait loci (mQTLs) were found distributed unevenly across the genome. Twenty‐four candidate genes were found to modulate the levels of different metabolites, of which two were functionally annotated by *in vitro* analysis to be involved in the synthesis and modification of flavonoids. Combining the correlation analysis of metabolite‐agronomic traits with the co‐localization of methylation quantitative trait locus (mQTL) and phenotypic QTL (pQTL), genetic relationships between the metabolites and agronomic traits were uncovered. For example, a candidate was identified using correlation and co‐localization analysis that may manage auxin accumulation, thereby affecting number of grains per spike (NGPS). Furthermore, metabolomics data were used to predict the performance of wheat agronomic traits, with metabolites being found that provide strong predictive power for NGPS and plant height. This study used metabolomics and association analysis to better understand the genetic basis of the wheat metabolism which will ultimately assist in wheat breeding.

## Significance Statement

This work performed a comprehensive metabolome analysis of kernels from wheat RILs, which, in conjunction with wheat genome data, provided a valuable resource for mQTL analysis, identification of unknown enzymes, and metabolite‐agronomic trait correlations.

## Introduction

Plants are highly enriched in specific metabolites that play important roles in the plant life cycle and mediate their interactions within the complex environments in which they live (Dixon and Strack, 2003; Saito and Matsuda, 2010; Peng *et al.*, 2017). Metabolomics aims to be the qualitative and quantitative analysis of all metabolites in a biological sample (Fiehn *et al.*, 2000), however current methodologies fall well short of this goal (Alseekh *et al.*, 2017). That said, combining metabolomics with genomics and transcriptomics has proven powerful in analyzing metabolic diversity and its underlying genetic variation, as well as in identifying numerous new genes and metabolic pathways (Tohge and Fernie, 2010; Fernie and Tohge, 2017; Alseekh and Fernie, 2018; Fang *et al.*, 2019). For example, hundreds of metabolic quantitative trait loci (mQTLs) have been detected in Arabidopsis, tomato, maize, and rice by linkage analysis (Lisec *et al.*, 2009; Matsuda *et al.*, 2012; Toubiana *et al.*, 2012; Alseekh *et al.*, 2015; Jin *et al.*, 2017), with the identification a large number of structural and regulatory genes involved in managing crop metabolite abundances. Using mQTL analysis, the complex metabolic pathways of plants can be better understood, with considerable advances being made in understanding the biosynthesis of glucosinolates in Arabidopsis and flavonoids in rice (Keurentjes *et al.*, 2006; Gong *et al.*, 2013).

Wheat (*Triticum aestivum L.*) is one of the most important crops worldwide. It provides *c.* 20% of the calories and 25% of protein consumed by humans (Matros *et al*., 2017). The metabolomics approach has been applied broadly to many crops, but remains limited to wheat. The latest report studied 76 metabolites from 135 winter wheat lines, allowing for a genome‐wide association study (GWAS) and revealing six distinct mQTL from the correlation of metabolite traits and associated single nucleotide polymorphisms (SNPs) (Matros *et al.*, 2017). So far, the largest scale analysis in wheat is that reported by Hill *et al. *(2015), who analyzed 558 metabolite and 10 agronomic trait quantitative trait locus to investigate the genetic relationship between metabolite levels and agronomic traits (Hill *et al.*, 2015). Given that the reference genome of wheat was released in 2018 (http://www.wheatgenome.org/), the subsequent development of high‐throughput genetic analysis techniques will likely prove a considerable basis for the metabolomics study of wheat.

Resolving the genetic basis underlying phenotypic traits has been an important goal in plant sciences since the times of Gregor Mendel. Many genes have been cloned and functionally verified via linkage and association analyses; however, in general, the mechanisms defining end‐phenotypes have yet to be elucidated (Huang and Han, 2014; Zuo and Li, 2014). The plant metabolome is often regarded as the bridge between the genome and phenome, since in its broadest sense the metabolome defines the phenotype (Luo, 2015; Fang *et al.*, 2016; Yang *et al.*, 2017), and its combination with the quantitative genetic analysis has greatly aided researchers inferring the genetic links between the plant metabolism and phenotypic variation (Lisec *et al.*, 2008; Fernie and Schauer, 2009; Carreno‐Quintero *et al.*, 2012; Wen *et al.*, 2014; Chen *et al.*, 2016; Zhu *et al.*, 2018). As such, metabolites could play an important role as biomarkers to predict complex agronomic traits, thus could allow for the rapid acceleration of the breeding processes, while at the same time lowering their costs (Meyer *et al.*, 2007; Riedelsheimer *et al.*, 2012; Xu *et al.*, 2017; Ghosh *et al.*, 2018).

In the current study, we analyzed 1260 metabolite features, of which we were able to structurally annotate 467 using a widely targeted LC‐MS/MS approach. We were subsequently able to localize 1005 high‐resolution mQTLs based on the Wheat660K high‐density SNP map, combining the latest wheat genome annotations (https://www.wheatgenome.org/). On the basis of this analysis, we were able to assign 18 candidate genes and verify two of them by *in vitro* expression studies. Additionally, the relationships between the mQTLs and a range of phenotypic QTL (pQTL) were investigated, revealing the possibility of predicting agronomic traits by using metabolic data. As a result, this study considerably improved both our knowledge of wheat metabolomics and its relationship to agronomic traits, providing a powerful tool for crop improvement.

## Results

### Metabolic profiling and broad‐sense heritability

**Figure 1:**
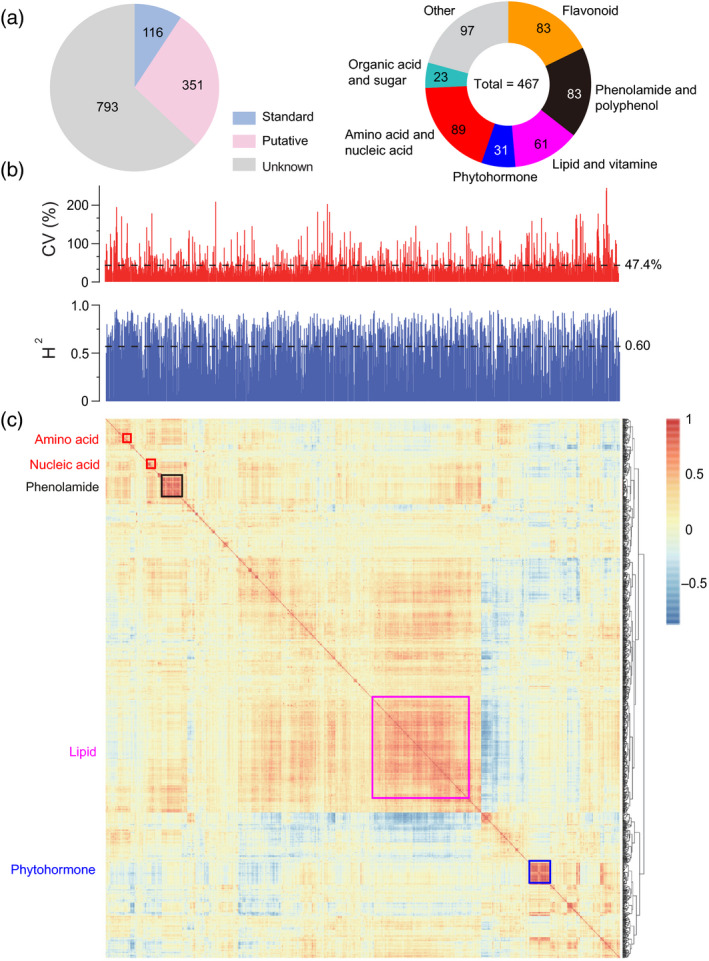
Metabolic profiling in wheat RIL population. (a) Number of detected metabolites and their classification. (b) Distribution of the values of coefficient of variation (CV) and broad‐sense heritability (*H* ^2^) of metabolic traits in the RIL population. *H* ^2^ was estimated using one‐way ANOVA, taking into account the variations between the three biological replicates as phenotypic variance derived from environmental factors. (c) Pairwise Pearson’s correlations are shown in a heat map, whereas metabolites are sorted according to correlation‐based hierarchical cluster analysis. The level of correlation is indicated by red (positive correlation) and blue (negative correlation).

Samples of the mature kernels of 145 recombinant inbred lines (RILs) derived from a cross between two elite wheat varieties, Kenong 9204 (KN9204) and Jing 411 (J411), were collected. The parental lines were highly varied in grain traits and spike characteristics with the aim to identify the major genes affecting the agronomic traits (Fan *et al.*, 2019; Zhao *et al.*, 2019). Using a high‐throughput LC‐MS/MS method, which was previously established to be widely targeted (Chen *et al.*, 2013), we detected and quantified 1260 distinct metabolite features from the mature kernels extracts of the inbred lines with three biological replicates (Tables S1 and S2). Of these metabolic features, 116 were structurally identified by direct comparison of their chromatographic and fragmental behaviours to those of the authentic standards, while 351 were putatively annotated by using previously described strategies (Chen *et al.*, 2013). Most annotated (and putatively annotated) compounds were flavonoids, phenolamides, polyphenols, lipids, vitamins, phytohormones, and their derivatives, amino acids and their derivatives, nucleic acids and their derivatives, organic acids, and sugars. Thus, we achieved a coverage of multiple important metabolic pathways (Figure 1a and Table S1).

The levels of metabolite accumulation varied widely across the lines, which allowed for an efficient analysis of their genetic architecture. Across the RIL population, these metabolites had an average genetic coefficient of variation (CV) of 47.4% (Figure 1b). There was, however, a considerable variation between compound classes with phenolamides and polyphenols, with a maximum average CV of 59.8% and a range from 13.6% for spermine to 194.5% for *N*′, *N*″‐Di‐*p*‐coumaroyl spermidine (Table S3). The distributions of broad‐sense heritability (*H* ^2^) of the metabolic traits demonstrated that over 56% of the metabolites displayed a heritability of above 0.6 (Figure 1b). Generally, among the annotated metabolites, the secondary metabolites exhibited higher *H* ^2^ (with the average of 0.63) than the primary metabolites (with an average of 0.58), and flavonoids exhibited the highest heritability (*H* ^2^ > 0.70) (Table S3). Thus, these data indicated that metabolite diversity was mainly influenced by heritable factors.

Metabolite profiling can elucidate the links between metabolic pathways (Toubiana *et al.*, 2012; Hill *et al.*, 2013; Matros *et al.*, 2017). Therefore, these metabolite correlations were analyzed using Spearman’s rank correlation, and a heatmap was constructed for all the detected metabolites. This revealed a more positive correlation (in red) than the negative correlations (in blue), as well as some densely correlated metabolite clusters (Figure 1c). For example, the coloured boxes in the upper‐left corner were mainly constituted of amino acids and their derivatives, nucleic acids and their derivatives, and phenolamides. The purple and blue boxes at the bottom represented the high positive correlation between lipids and phytohormones and their derivatives, respectively (Figure 1c). These closely related metabolites are most likely either the same type of molecules or molecules belonging to the same biochemical pathway. This phenomenon was confirmed by the correlation coefficients illustrated in Figure S1. Most amino and nucleic acids were present in one tight metabolite cluster, while flavonoids were relatively dispersed, although they were more closely related to each other than to other substances (Figure S1). The lipids, polyphenols, and phenolamides were found across several large clusters, suggesting that these metabolites were involved in multiple metabolic pathways and potentially play different physiological roles (Figure S1).

### mQTL mapping in mature kernels using a high‐density SNP map

**Figure 2:**
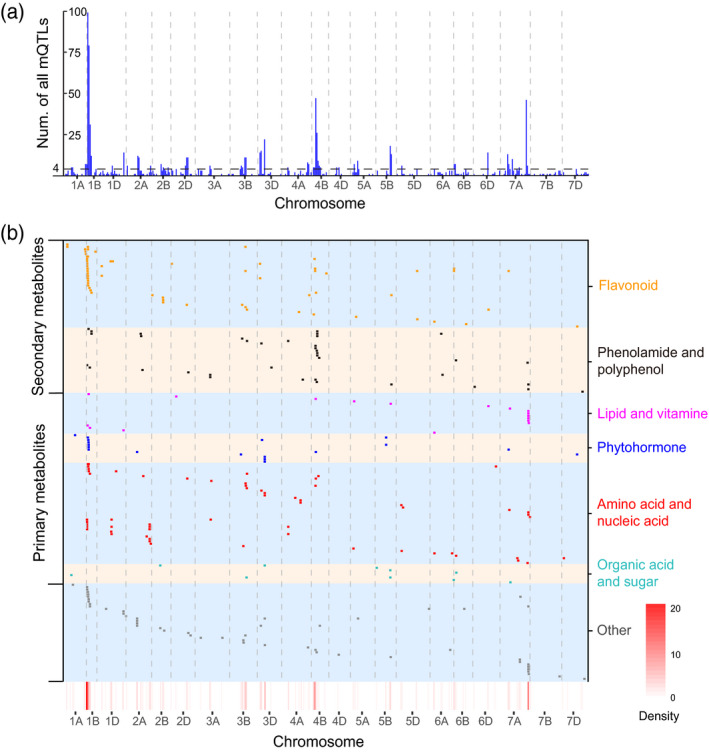
Chromosomal distribution of metabolic QTLs (mQTLs) identified. (a) Chromosomal distribution of all mQTLs (1005) and mQTL hotspots. The horizontal dashed line indicates the threshold for mQTL hotspots, represented by the maximum number of mQTLs expected to fall into any interval by chance alone with a genome‐wide *P* = 0.01. The interval size is 10 cM. (b) Distribution of mQTLs of 467 known metabolites on chromosomes. Each row represents the QTL mapping of single metabolic traits. Metabolites from different chemical groups are marked by distinct colours. The *x*‐axis indicates the genetic positions across the wheat genome. The heat map under the *x*‐axis illustrates the density of QTL across the genome. The window size is 10 cM.

The RIL population used in this study was fine mapped using the Affymetrix Wheat660K SNP array as a probe, as described previously (Cui *et al.*, 2017). Based on this high‐density map, 1005 mQTL from 746 metabolites (out of the total 1260 metabolites) were reproducibly mapped from three environments of the RIL population [logarithm of odds (LOD) ≥2.5] (Figure 2a and Table S4). Among these, approximately half of QTL (493) were concentrated on the B genome (Figure S2a), with the number of flavonoid‐related QTL (61) being the highest compared with other categorized metabolites, followed by amino acids, nucleic acids, and their derivatives (Figure S2b).

A chi‐squared test revealed the random distribution of all mQTLs (1005 mQTLs) across the genome (*X* ^2^ = 207.1, *P* < 2.2e‐16; Table S5). Remarkably, we observed 68 hotspots in the genome, mainly localized on the 1B, 4B, and 7A chromosomes, but especially in 1B (Figure 2a). The hotspot regions with significant enrichment of mQTL were likely to have major regulatory genes affecting multimetabolic traits. Flavonoid‐ and phenolamide‐related hotspots were found on 1B and 4B, respectively, while five lipid metabolite‐related QTLs co‐located to 7A: 240.0–240.8 cM (Figure 2b). Conversely, the number of mQTLs detected across several chromosomes, such as chromosomes 3A and 4D, was significantly less than expected (Figure 2a and Table S5).

For each metabolite the number of mQTLs varied from one to six with 201 metabolites having at least two mQTLs (Table S4). However, several metabolites were influenced by a single major mQTLs (Table S4). For instance, a QTL for the level of n16920 (a polyphenol putatively annotated as hydroxycinnamoyl‐glyceric acid) was mapped on chromosome 2A between 735.0 and 735.1 Mb (LOD = 15.3), it explained 33.4% of the phenotypic variance; another QTL for mr1093 (tricin *O*‐malonylhexoside) was mapped on chromosome 2B between 665.2 and 666.4 Mb (LOD = 11.9), explained 31.2% of the phenotypic variance (Table S4). These results suggested that a single gene, rather than an epistatic interaction, was directly involved in the synthesis of the metabolite.

Each mQTL explained 0.8–53.1% of the observed phenotypic variation, with a mean value of 13.3%, with 263 loci associated with a phenotypic variation of over 15% (Figure S3a and Table S4). Among them, the phenotypic variation explained (PVE) of the secondary metabolites QTLs (average PVE, 14.0%) was generally greater than that of the primary metabolites (average PVE, 11.9%) (Figure S3b). Different PVEs could partly reflect the different genetic structures between the primary metabolism (central metabolism) and secondary metabolism. The full lists of mQTL in Table S4 represent an important resource for further functional verification and subsequent application in trait‐oriented studies.

### Identification of candidate genes underlying mQTLs

The high resolution of the mQTLs facilitated the assignment of metabolite candidate genes. We screened a series of candidate genes by integrating the structure of the compounds, known biosynthetic pathways, and wheat genome annotations (Tables 1 and S4). In the vicinity, *TraesCS5D01G028100* which encodes a putative amino acid permease family protein, was assigned as a candidate due to its high similarity to both functionally annotated Arabidopsis and the rice genes *AtPUT2* and *OsPAR1* (70 and 87% identity at the amino acid level, respectively; Li *et al.*, 2013; Dong *et al.*, 2016). Moreover, multiple flavonoids were mapped to a single locus (588.7–593.5 Mb on chromosome 1A; Table S4), and two genes in the interval ‒ *TraesCS1A01G442200* and *TraesCS1A01G442300* ‒ shared a high identity (70 and 78% identity at the amino acid level, respectively) to a rice flavonoid 3′‐hydroxylase encoding gene *OsF3’H* (Shih *et al.*, 2008). A further two candidate genes were chosen from the list and verified by *in vitro* expression analysis, as described below.

**Table 1:** Summary of candidate genes for metabolic quantitative trait loci (mQTLs)

Metabolite	Chr	LOD	PVE^a^ (%)	Interval (Mb)	Candidate gene	Annotation
Chrysoeriol 7‐*O*‐rutinoside	1A	9.7	13.2	12.2–13.9	*TraesCS1A01G032300.1*	Chalcone synthase
*C*‐pentosyl‐luteolin *O*‐hexoside	1A	13.5	28.5	588.7–593.5	*TraesCS1A01G442300.1*	Flavonoid 3′‐hydroxylase
*N* ^6^‐benzyladenine‐9‐glucoside (BA9G)	1B	9.8	25.6	464.1–487.7	*TraesCS1B01G272500.1*	Glycosyltransferase
*N*‐Acetyltryptophan	1B	9.1	23.6	464.1–487.7	*TraesCS1B01G280400.1*	Transferase
*N*‐Methyl histamine	1B	3.6	10.5	558.6–562.7	*TraesCS1B01G332100.1*	Methyltransferases
*C*‐Pentosyl‐apigenin *O*‐feruloyl hexoside	1D	7.7	17.1	7.0–8.5	*TraesCS1D01G020700.1*	Transferase
Tricin 7‐*O*‐hexosyl‐*O*‐xyloside	1D	11.3	29.9	404.1–415.4	*TraesCS1D01G319100.1*	Glycosyltransferase
*N*′, *N*″‐Di‐*p*‐coumaroylspermidine	2A	4.9	11.2	726.4–728.6	*TraesCS2A01G490000.1*	Transferase
Apigenin 7‐*O*‐rutinoside	2B	4.9	10.3	5.6–7.2	*TraesCS2B01G012000.1*	Glycosyltransferase
3′,4′,5′‐Tricetin *O*‐rutinoside	2B	13.2	29.6	654.4–654.0	*TraesCS2B01G459900.1*	Glycosyltransferase
Tricin *O*‐malonyl hexoside	2B	11.9	31.2	665.2–666.4	*TraesCS2B01G472400.1*	Transferase
Tricin	2D	4.8	14.3	624.4–624.8	*TraesCS2D01G530600.1*	Chalcone synthase
Tricin	3B	4.0	10.7	462.0–468.5	*TraesCS3B01G290200.1*	Glycosyltransferase
2‐Methyladenosine	4A	4.2	8.4	17.6–18.3	*TraesCS4A01G024100.1*	Methyltransferase
Tricin 5‐*O*‐hexosyl‐*O*‐hexoside	4A	13.1	17.4	628.6–628.7	*TraesCS4A01G350700.1*	Glycosyltransferase
Chrysoeriol 6‐*C*‐hexoside	4B	8.4	13.6	13.1–25.6	*TraesCS4B01G021600.1*	Chalcone synthase
*N*‐Feruloylputrescine	4B	5.7	14.5	26.4–27.5	*TraesCS4B01G026800.1*	Transferase
*N*‐Salicyloylserotonin	4B	2.9	7.3	623.8–625.8	*TraesCS4B01G336000.1*	Transferase
Apigenin 7‐*O*‐rutinoside	5B	2.9	8.0	549.6–562.5	*TraesCS5B01G383300.1*	Chalcone synthase
Saccharopine	5D	5.5	14.6	27.8–28.0	*TraesCS5D01G028100.1*	Amino acid permease
*N*′, *N″*‐Di‐Feruloylspermidine	6A	2.9	6.4	442.0–452.6	*TraesCS6A01G242600.1*	Transferase
Caffeoyl *O*‐hexoside	6A	3.4	7.1	561.9–562.4	*TraesCS6A01G327100.1*	Glycosyltransferase
Chrysoeriol	6D	3.9	9.3	419.3–425.0	*TraesCS6D01G310700.1*	Flavonol synthase
Apigenin 6‐*C*‐glucoside	7D	13.6	2.4	100.6–101.5	*TraesCS7D01G152300.1*	Dihydroflavonol‐4‐reductase

Chr, chromosome; LOD, logarithm of odds.

^a^ PVE (%), Variation explained by the QTL. More information is listed in Table S4.

**Figure 3:**
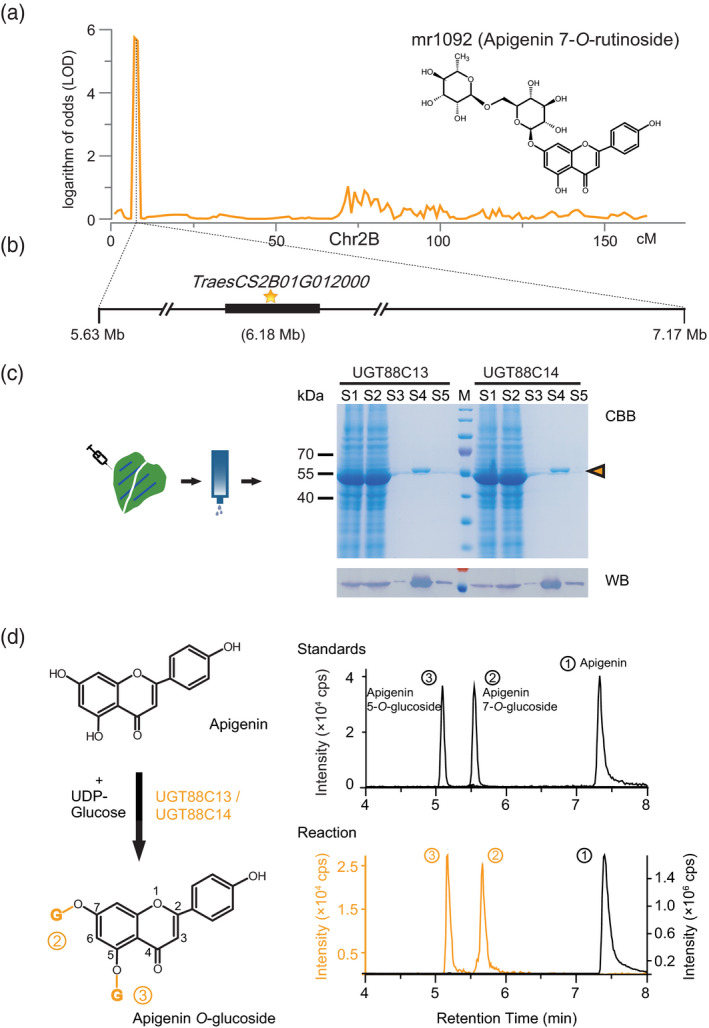
Functional annotation of candidate gene *TraesCS2B01G012000* (a) LOD curves of QTL mapping of the mr1092 (Apigenin 7‐*O*‐rutinoside) accumulation on chromosome 2B. (b) Gene model of *TraesCS2B01G012000*. The black box represents the coding sequence. (c) Candidate gene encoded proteins were transiently expressed in *N. benthamiana* followed by a *Strep*II purification. Samples (5 µl) were taken at different stages of the purification. Lanes S1 to S5 are total soluble proteins; proteins not bound; last wash fraction; elution fraction; and proteins left on the matrix after elution, respectively. The arrowhead indicates the purified protein. CBB, Coomassie Brilliant Blue stain; WB, western blot. (d) Enzymatic reaction by the purified proteins. The structures of the substrates and products (left) and the chromatograms of the standards and the biochemical reaction.

The mQTL of mr1092 (apigenin 7‐*O*‐rutinoside) was mapped to the interval 5.6–7.2 Mb on chromosome 2B (Figure 3a). In this region, a gene was annotated as a putative glycosyltransferase ‒ *TraesCS2B01G012000* ‒ whose encoded protein displayed a 49.1% identity to rice UGT706D1 (Figure S4). The coding sequence was cloned from Chinese Spring (CS) into a *Strep*II‐tagged vector under the control of the 35S promoter and expressed in *N. benthamiana* (Figure 3c). Apigenin and tricin were tested as co‐substrates alongside UDP‐glucose and the purified protein, revealing that it accepted apigenin but not tricin (Figure 3d). More substrates were tested, with the results presented in Table S6. The protein was registered under the name of UGT88C13 (by the UGT Committee). When cloning this target gene from two parental lines, we noted difficulty in its amplification from variety J411. Several primer pairs were used and a positive result was only obtained from line KN9204 and CS (Figure S5). Thus, it is very likely that a sizable sequence change occurred in J411, or the loss of the gene during the evolution or domestication of J411. The translated protein from KN9204 (named UGT88C14) was expressed, extracted, and purified from *N. benthamiana* (Figure 3c) and showed a similar coding sequence and the protein activity of CS (Table S6 and Figure S6). This result demonstrated the glucosyltransferase activity of the candidate, thus accounting for the variant accumulations of glycosylated apigenin in the RIL population.

Similarly, another flavonoid‐related gene was targeted via mQTL mr075 (3′,4′,5′‐tricetin *O*‐rutinoside), and only three genes were found to be located within the interval. One of the three genes, *TraesCS2B01G459900*, was annotated as a glycosyltransferase, similar to the rice UGT706C1 (52.1% identity at the amino acid level). Therefore, we cloned this gene from CS. Although activity was detected (Figure S7a, b), we noted that the two parental lines had the same coding sequence. Therefore, qRT‐PCR was performed to determine the relative expression levels. The results demonstrated that the relative expression of the target gene in J411 was *c.* 10 times greater than its expression in KN9204 from the tissue harvested during the second week of grain filling (Figure S7c). This observation is in line with the fact that the glycosylated product accumulated at a high level in J411 than in KN9204.

### Correlations between agronomic traits and metabolites

**Figure 4:**
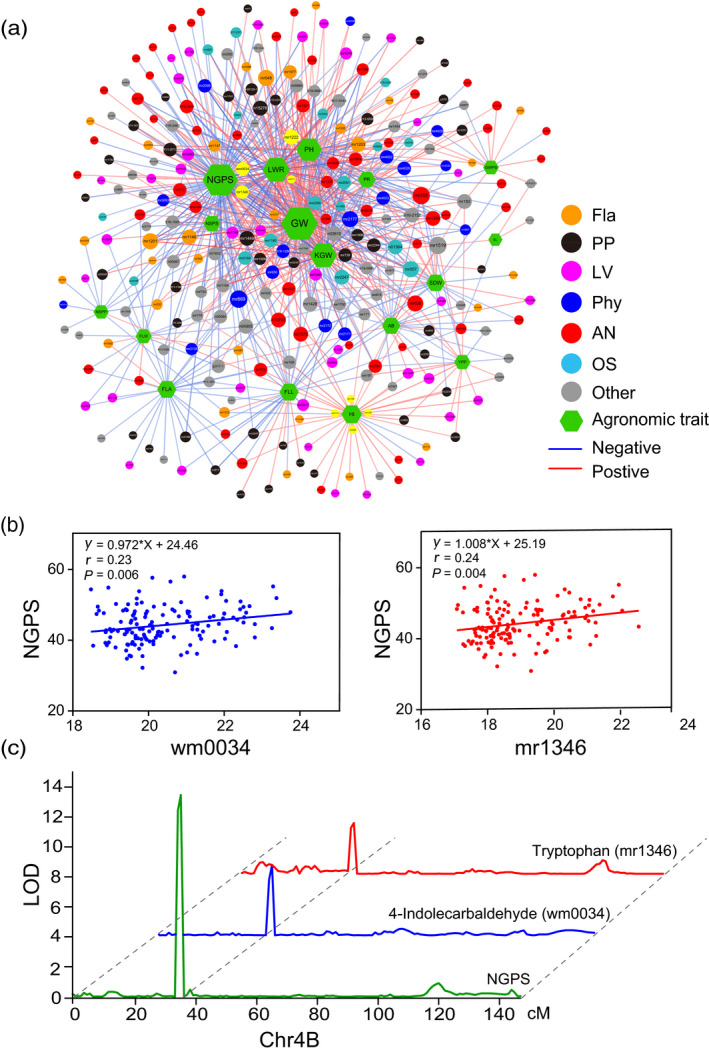
Association network visualization of co‐detected metabolite‐agronomic traits and dissection of a candidate gene associated with number of grains per spike (NGPS). (a) Association analysis of 467 annotated metabolites with 17 agronomic traits. Co‐detected metabolites and agronomic traits are represented as nodes, and their correlation coefficient values as edges. The absolute values of the Pearson’s correlation coefficient values above the threshold (*P* < 0.01) are shown. Different colours represent different classes of metabolites. Circles and green hexagons are represented as metabolites and agronomic traits, respectively, where the size of the shape represents the number of associations. The level of correlation is indicated as red (positive correlation) or blue (negative correlation). The intensity of the colour indicates the correlation, where a darker colour denotes a stronger correlation. The yellow circles indicate metabolites that are significantly associated with the co‐localization of close agronomic traits. PR, panicle rate; YPP, yield per plant; NSPP, number of spikes per plant; AB, aboveground biomass; SDW, straw dry weight; LWR, length width ratio of seed; GW, grain width; NSPS, number of spikelets per spike; FLW, flag leaf width; FLA, flag leaf area; FLL, flag leaf length; KGW, kilo‐grain weight; HI, harvest index; NGPS, number of grain per spike; GWPS, grain weight per spike; SL, spike length; PH, plant height. (b) Correlation analysis between two metabolites (wm0034, 4‐indolecarbaldehyde; mr1346, tryptophan) and NGPS. (c) LOD curves of QTL mapping for number of grains per spike, wm0034 (4‐indolecarbaldehyde), and mr1346 (tryptophan) levels on chromosome 4B. Green, number of grains per spike; Blue, 4‐indolecarbaldehyde; Red, tryptophan.

The 17 agronomic traits of this RIL population were previously obtained in three independent harvests, as described in Fan *et al. *(2019) and Zhang *et al. *(2017). To analyze the relationships between the changes of metabolites and plant morphology, we started by determining the CV of the 17 agronomic traits. The CV was found to range from 3.8 to 15.7%, with an average *H* ^2^ of 0.61 (Figure S8), which suggested the potential of a significant genetic contribution and the artificial selection of beneficial agronomic characteristics. A metabolite‐agronomic trait association network was subsequently constructed, consisting of 467 annotated metabolites and 17 agronomic traits (Figure 4a). Then, 754 significant correlations (*P* < 0.01) were determined, with an approximately similar number of positive and negative correlations (Figure 4a and Table S7) and 264 (56.5%) metabolites correlated with at least one agronomic trait (Table S7). For example, mr869 was associated with eight agronomic traits (Table S7). Flavonoids, amino acids, nucleic acids, lipids, phenolamides, and polyphenols were significantly correlated with 13 of the agronomic traits (Figure 4a and Table S7), which indicated that the metabolites were involved in the formation of the agronomic traits. In line with previous results (Hill *et al.*, 2013), we found that the correlations between metabolic and agronomic traits was not as tight as the correlations among metabolic traits (Tables S2 and S7).

Among the agronomic traits, grain width (GW), harvest index (HI), and kilo‐grain weight (KGW) were mainly positively correlated with the annotated metabolites. However, flag leaf‐related traits (flag leaf length, FLL; flag leaf width, FLW; flag leaf area, FLA) and spike‐related traits (number of spikes per plant, NSPP; number of grains per spike, NGPS; number of spikelets per spike, NSPS) were mostly negatively correlated with the annotated metabolites (Figure 4a and Table S7). Interestingly, from the correlation data, we found that leaf traits (FLL, FLW, FLA) and grain traits (KGW; grain width, GW; length width ratio of seed, LWR) were significantly correlated with 56 and 141 metabolites, respectively (Figure 4a), indicating that the formation of grain traits may be more complex than that of the leaf traits. Moreover, 54 metabolites were significantly correlated with the three grain traits (Table S7), suggesting that adjusting the contents of these metabolites could be used as a strategy to improve the yield and quality of grain.

### Colocalization of mQTLs and pQTLs

To further investigate the relationship between agronomic traits and metabolites, we used the agronomic trait data for pQTL analysis. As a result, 97 pQTLs were identified for the 17 agronomic traits, which were mainly located on chromosomes 2D and 4B, with the most QTL (11) detected for plant height (PH trait; Table S8). PVE ranged from 1.9 to 37.6%, with an average of 8.3%, which was significantly lower than the average PVE of the mQTLs (13.3%) (Tables S4 and S8).

Next, the relationship between mQTL and pQTL was analyzed. Approximately half of the pQTLs (48) were found to overlap with the mQTL; in total, 369 mQTLs representing 252 metabolic features (including 61 annotated metabolites) colocalized with pQTL (Table S9). The most colocalized pQTL metabolites were mr1548 and mr2801 (unknown), followed by mr107 (chrysoeriol 6‐*C*‐hexoside) and mr1203 (methylluteolin *C*‐hexoside), which colocalized with six pQTLs covering five agronomic traits (Table S9). In the genome, several intervals were found that influenced the 10 abovementioned metabolites, while simultaneously affecting more than two agronomic traits; these intervals were mainly on chromosomes 1B and 4B. For example, the pQTL on chromosome 4B at the interval 23.7–30.9 Mb for LWR and NGPS was colocalized with 42 mQTLs.

Interestingly, the metabolites that colocalized with agronomic traits at the same time significantly correlated with the agronomic traits (Tables S7 and S9), indicating that the related metabolites affect the agronomic traits, or vice versa. For example, the level of metabolite mr1159 (ferulic acid, yellow circle) and three flavonoids (mr1114, *C*‐hexosyl‐apigenin *O*‐*p*‐coumaroylhexoside; n03958, tricin 7‐*O*‐hexosyl‐*O*‐xyloside; mr1120, *C*‐hexosyl‐chrysoeriol *O*‐*p*‐coumaroylhexoside) were correlated with the HI and colocalized at chromosome 1B (Table S9). The same was observed between mr1222 (*C*‐hexosyl‐chrysoeriol 7‐*O*‐hexoside) and PH, as well as n04711 (pyranose derivative) and the seed length width ratio (Figure 4a and Table S9). Intriguingly, mr1346 (tryptophan) and wm0034 (4‐indolecarbaldehyde), two metabolites involved in auxin synthesis, were significantly associated with NGPS and localized to a similar region of chromosome 4B (Figure 4b, c). According to the common PCR‐markers (approximate physical location, determined by flanking BLAST search) and the physical position of the SNP markers, one candidate protein encoded by *TraesCS4B01G155000* was annotated as an auxin‐repressed/dormancy‐associated protein (Figure 4c). Further identification of related genes could aid in the cloning of QTLs that affect these agronomic traits, as well as improve our understanding of the genetic structure of complex agronomic traits.

### Agronomic trait prediction using metabolic data

**Figure 5:**
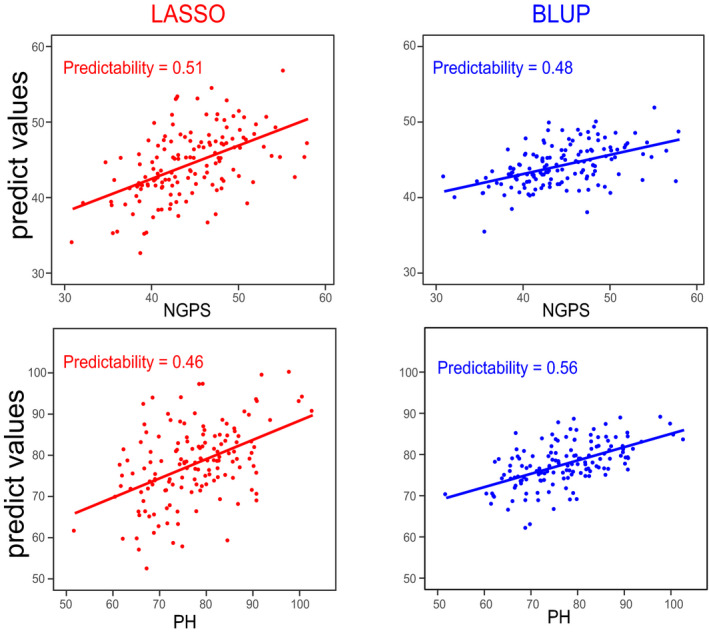
Metabolic data used to predict plant height (PH) and number of grains per spike (NGPS) based on two models. The BLUP and LASSO models were used to predict the plant height and number of grains per spike, respectively. Right: BULP prediction result. Left: LASSO prediction result. The x‐axis indicates the predictive value of agronomic traits and the y‐axis indicates phenotypic observations. The image was made using R (http://www.r‐project.org/).

Based on BLUP and LASSO models, we combined large‐scale metabolic data (1260 metabolite features) to predict 17 agronomic traits. The average predictability of the 17 agronomic traits by BLUP and LASSO were 0.26 and 0.27, respectively, with the highest predictabilities in trait PH (0.51, average of two models) and trait NGPS (0.49) (Figure 5 and Table S10).

The LASSO method detected 82 and 98 metabolite features, including phytohormone derivatives, sugars, and organic acids, among others, and resulted in significant effects on NGPS and PH (*P* < 0.05), as listed in Table S10. Among the metabolites, mr169 (betaine, *P* = 0.036) and S19‐0168 (unknown, *P* = 0.007) were found to have the greatest positive effects on PH and NGPS prediction, respectively (Table S10). The metabolite mr355 (2′‐deoxyinosine‐5′‐monophosphate, *P* < 0.05) displayed a high predictive effect on both PH and NGPS. These metabolites, which have significant effects on prediction, could contribute to the improvement of wheat breeding.

## Discussion

The combination of metabolomic and genomic approaches has been widely used to determine the genetic basis of metabolic diversity. However, most studies to date have only focused on Arabidopsis, tomato, rice, and maize (Lisec *et al.*, 2009; Chen *et al.*, 2014; Wen *et al.*, 2014; Luo, 2015; Alseekh *et al.*, 2015). Recent advances in the development of the omics toolbox of wheat are, however, paving the way for a deeper understanding of the metabolic diversity of this crop (Appels *et al.*, 2018). In this study, metabolomics was combined with high‐resolution genotyping of a RIL population to analyze gene‐metabolite and metabolite‐agronomic trait associations.

### Metabolomics and mQTLs

The detection of metabolites is the basis for studying their genetic variation. In this study, 1260 metabolites were obtained by widely targeted LC‐MS/MS, resulting in the identification of the chemical structure of 467 metabolites. The results obtained in this study represent a considerable advance with regards to the detection of metabolites compared with previous wheat metabolome studies (Hill *et al.*, 2015; Matros *et al.*, 2017). Here, important compound classes were included, such as polyphenols and flavonoids, which are essential in plant biotic/abiotic stresses and have multiple impacts on human health (Winkel‐Shirley, 2002; Zhang and Tsao, 2016). The primary metabolites generally displayed strong correlations, such as amino acids, nucleic acids, phytohormones, and lipids (Figures 1c and S1). This is consistent with previous studies in rice, wheat, and tomato (Matsuad *et al.*, 2012; Sauvage *et al.*, 2014; Matros *et al.*, 2017). Meanwhile, metabolite correlations were found, some of which showed strong correlations, such as phenolamides and flavonoids (Figures 1c and S1). The correlation analysis among metabolites not only reflects the relationships of known molecules, but also the relationship between unknown molecules and known molecules, providing an important resource for future efforts in the identification of unknown metabolites and pathways.

Based on the Wheat660K high‐density genetic map‐based linkage analysis, 1005 mQTLs were found randomly distributed across the wheat genome (Figure 2 and Table S4). Among them, many high‐resolution mQTLs were reported. Moreover, the occurrence of mQTL contributing to the levels of many different metabolites was observed, and 68 hotspots were identified from the kernels, the majority of which were found on chromosomes 4B and 1B (Figure 2). These hotspots were also detected in the previous studies on Arabidopsis, rice, tomato, and maize (Keurentjes *et al.*, 2006; Matsuda *et al.*, 2012; Gong *et al.*, 2013; Wen *et al.*, 2015; Knoch *et al.*, 2017), demonstrating that this phenomenon is common and important. These findings indicate that many metabolites can be influenced by the manipulation of small genomic regions, suggesting that manipulating metabolism by breeding is tangible (Saito and Matsuda, 2010).

### Candidate genes and pathway analysis

One important advantage of this study compared with earlier studies is that the availability of the hexaploid wheat genome allowed for candidate gene identification directly from QTL mapping (Appels *et al.*, 2018). In this study, 24 candidate genes were assigned according to the annotation and study of the corresponding genes in model plant species (Tables 1 and S4). Two of the candidate genes from mQTL mapping were verified by recombinant protein activity assays or mRNA expression analysis (Figures 3 and S7). For the first candidate, the protein was verified to be a UDP‐glycosyltransferase (UGT) that could glycosylate different oxygen atom positions of the flavonoid A and B rings. According to our enzymatic tests (Table S6), this UGT accepted apigenin, luteolin, kaempferol, and quercetin, but not flavonoids in which the B ring was methylated; it preferred the position 7‐OH for the addition of glucose above the 4′‐OH. This phenomenon of multiple‐position glycosylation was previously observed in rice (Ko *et al.*, 2008). However, Peng *et al. *(2017) demonstrated mostly position‐specific glycosylation, including two major flavone UGTs responsible for the glucosylation of the 7‐OH and 5‐OH groups of rice flavones (*OsUGT706D1* and *OsUGT707A2*, respectively). A phylogenetic tree including our verified *Ta*UGTs and the other known UGTs was created for its classification. The result show that the *Ta*UGTs is classified in the UGT88C subgroup, which is not well identified (Figure S6). According to our result, this subgroup is likely to function mainly in flavanol 7‐*O*‐glucosyltransferases, and does not exclude glycosylated 5‐OH and 3′‐OH groups simultaneously, depending on the modifications of the rings. Unlike the first candidate, the second verified gene, *TraesCS2B01G459900*, encoding UGT706E7 (by the UGT Committee), played a role in the variations of the corresponding metabolite accumulation by the expression levels during grain filling (Figure S7). The purified protein showed activity to substrate 3′,4′,5′‐*O*‐trimethyltricetin with the glycosyl donor of UDP‐glucose (Figure S7b), and minor activity to substrates chrysoeriol indicating that the protein preferred all 3′,4′,5′‐position methylated flavonoids.

The genes in the candidate list were associated with multiple metabolic pathways, including those involving flavonoids, phenolamides, and amino acids (Tables 1 and S4). Flavonoids accounted the largest major proportion of the metabolites classified. A putative network of wheat flavone‐ and flavonol‐related metabolic pathways is shown in Figure S9. The genes assigned were either described in our results (red) or their homologues were previously reported (blue). For example, *TraesCS1D01G020700*, which was mapped by mr1120 and mr1112 (Table S4), had a considerable PVE and was about 300 kb away from the confidence interval. Its homologue in rice (*LOC_Os02g28170*), encoding OsMAT‐2, was verified to be a flavonoid malonyltransferase using a recombinant protein assay (Kim *et al.*, 2009). Its corresponding homologue in maize (*GRMZM2G387394*), which encodes AAT1, was the first anthocyanin acyltransferase characterized in a monocot species, which analyzed by mutation phenotype (Paulsmeyer *et al.*, 2018). Based on these findings, the *TraesCS1D01G020700* gene was assigned. These assigned genes have not yet been reported in common wheat, however, further evidences are required to verify their functions. The same is true for the other genes in the candidate list (Table S4).

The large‐scale and high‐resolution nature of the mQTLs in this study benefited from the high coverage, sensitivity, and accuracy of the metabolomics method used and the high density of the SNP markers (Chen *et al.*, 2013; Cui *et al.*, 2017). In future studies, the hundreds of loci identified in this study could be further verified and characterized, which would help dissect the molecular basis of metabolic variation and elucidate new functional proteins and metabolic pathways in the common wheat.

### Associations between metabolic and agronomic traits

Metabolites are considered as the bridge that links the genome with the phenome. As such, studying the phenotypic‐ and metabolic‐related properties greatly reflects the value of this bridge (Luo, 2015). In a QTL analysis of potato, Carreno‐Quintero *et al. *(2012) found that metabolites were colocalized with starch‐ and cold sweetening‐related traits. Chen *et al. *(2016) demonstrated that trigonelline positively affects GW by elongating the G2 phase and the duration of the whole cell cycle. Further studies have shown that analyzing the colocalization of metabolites‐agronomic traits helps to infer genetic links in maize and tomato (Toubiana *et al.*, 2012; Wen *et al.*, 2015). In this study, mQTL analysis revealed that wm0034 (4‐indolecarbaldehyde) and mr1346 (tryptophan) were colocalized, both of which are found in the tryptophan pathway and are involved in auxin biosynthesis. Using network analysis (Figure 4a), NGPS was found to be significantly correlated with these two metabolites (*P* < 0.01). Moreover, the loci corresponding to NGPS in the pQTL analysis was found to be colocalized with the abovementioned mQTL on chromosome 4B (Figure 4c). In this colocalized region, a coding sequence ‒ *TraesCS4B01G155000*, Chr4B: 27.6 Mb ‒ for auxin‐repressed/dormancy‐associated proteins were found in the wheat genome annotation. Previous studies have showed protein function as an inhibitor of auxin accumulation (Zhao *et al.*, 2014; Souza *et al.*, 2019). For example, Reddy and Poovaiah (1990) showed that a high transcript abundance of the auxin‐repressed gene *SAR5* was correlated with the cessation of fruit growth in strawberries, or that the overexpression of *BrARP1* (encoding auxin‐repressed protein 1) or *BrDRM1* (encoding dormancy‐associated protein 1) led to smaller plants and shorter siliques (Lee *et al.*, 2013). Therefore, this candidate could disturb the plant yield by negatively adjusting auxin levels and the NGPS. Whether metabolite changes are the cause of changes in agronomic traits will require further experimental evidence and corresponding analysis.

The abovementioned strategies have been previously used to elucidate the relationship between metabolomics and agronomic traits and the formation mechanism of phenotypic traits (Wen *et al.*, 2014; Chen *et al.*, 2016). Given the limitations of the bi‐parental populations, it is important to note that high‐throughput metabolomics analysis could be used in natural populations exhibiting rich genetic variation for genome‐wide association studies (GWAS) to accelerate the functional genomics (Chan *et al.*, 2011; Li *et al.*, 2012; Angelovici *et al.*, 2013; Chen *et al.*, 2014; Sauvage *et al.*, 2014; Matsuda *et al.*, 2015; Tieman *et al.*, 2017; Wu *et al.*, 2018). This type of approach is likely to prove highly effective in wheat studies in the future.

### Prediction of agronomic features

Genomic selection (GS) is more efficient than traditional molecular marker‐assisted selection (MAS) methods in molecular breeding. With the development of high‐throughput sequencing, as well as transcriptome and metabolome technologies, multi‐omics data have been used to predict complex agronomic traits, with great progresses being made in crop studies (Wang *et al.*, 2016; Xu *et al.*, 2017; Kremling *et al.*, 2018). In this study, we used the BLUP and LASSO methods to demonstrated that the predictability of yield‐related traits (PH and NGPS) reached 0.56 and 0.51, respectively (Figure 5). This result is comparable with previous studies, including Riedelsheimer *et al. *(2012) and Xu *et al. *(2016). Xu *et al. *(2016) used 1000 metabolomics features data from 210 RILs to effectively predict KGW and other traits, with an average predictability for KGW of 0.55, using BLUP and LASSO.

The LASSO model is able to effectively screen more than one thousand metabolites and select a limited number of the metabolites that have a major effect in the prediction of phenotypes, as shown in this study. To compare the use of metabolic data and genotypic data for prediction, the same prediction was performed by using genotypic data (Figure S10). Using LASSO, metabolic features were found to have a higher prediction value for NGPS and PH (0.51 and 0.46; Figure 5) compared with the prediction value using genotypic data (0.47 and 0.44; Figure S10). However, these values were reversed under the BLUP model, which correlates with the findings reported by Xu *et al. *(2016) and Riedelsheimer *et al. *(2012). When the number of metabolites is increased to the thousands or tens of thousands, or is combined with other omics data, such as transcriptome and genomic data, the power of the predictivity should be improved. As such, we postulate that these high‐effective metabolite features are important in biomarker‐assisted breeding, and may allow for accelerated plant breeding by providing earlier generation selection.

## Experimental procedures

### Plant materials and growth conditions

An RIL obtained from a cross between KN9204 and J411 (denoted by KJ‐RIL) was used in this study. In this study, 145 lines from the KJ‐RIL population were used for metabolome analysis. The plants were grown at Yantai in Shandong Province, China (121°35′E, 37°52′N). A randomized block design with two replications was used during the 2016–2017 and 2017–2018 cropping seasons, with 40 seeds hand‐planted in each row of a two‐row plot with 2 m long rows spaced 0.25 m apart. All lines were self‐pollinated, and the field experiment was carried out in accordance to the standard agronomic wheat management practices. All material planting conditions have been previously described in detail (Zhao *et al.*, 2019). For each line, spikes from five plants were harvested at the same maturity and bulked. Twenty mature dry kernels for each line were selected for the metabolic profiling analysis of each environment.

### Metabolite analysis by LC‐MS/MS

Mature wheat kernels were homogenized comminuted (29 Hz, 50 sec) using a tissue grinder (Schwingmühle Tissue Lyser II, Germany) for 50 sec at 29 Hz. For each sample, 100 mg of dry powder was weighed, mixed with 1.0 ml of 70% methanol containing 0.1 mg/l acyclovir (internal standard), vortexed, and extracted for 10 h at 4°C. This was followed by centrifugation at 9500 ***g*** for 10 min, after which the resulting supernatant passed through a 0.22 µm organic filter (SCAA‐104; ANPEL, China). The samples were analyzed using an LC‐ESI‐MS/MS system (HPLC) (Shim‐pack UFLC SHIMADZU CBM20A system, 5500 Q TRAP; Applied Biosystems, Framingham, MA, USA). A stepwise multiple ion monitoring‐enhanced product ion was used to construct the MS2T library, as previously described (Chen *et al.*, 2013). To facilitate the identification of the metabolites detected in our study, accurate m/z for each precursor ions was obtained using a time‐of‐flight mass spectrometry platform (HPLC, Shim‐pack UFLC SHIMADZU CBM20A system, Triple TOF 5600; Applied Biosystems). The quantification of the metabolites was carried out using a scheduled multiple reaction monitoring (MRM) method, as described previously (Chen *et al.*, 2013). The scheduled MRM algorithm was used with an MRM detection window of 90 sec and a target scan time of 1.0 sec in Analyst 1.5 software.

### Statistical analysis

The metabolite data were log_2_‐transformed for statistical analysis to improve normality. Broad‐sense heritability (*H* ^2^) was calculated using one‐way ANOVA with three biological replicates to determine the environmental effects (Visscher *et al.*, 2008). The values of the CV were calculated for each metabolite (using the average of the three biological replicates of untransformed data) and agronomic trait (using the average of the 3 years of data) expressed as S/A, where S and A represent the standard deviation and the average of metabolite and agronomic trait in the population, respectively. Pearson’s correlation and the statistical significance between traits were estimated using programs housed in R (http://www.r‐project.org/). Visualization correlation networks were constructed using Cytoscape 3.7.0 (Smoot *et al.*, 2010).

### QTL mapping and mQTL hotspot identification

A high‐density genetic map was constructed for the KJ‐RILs using the wheat‐660K SNP array (Cui *et al.*, 2017). The QTL analysis of each trait (three biological replicates metabolite and the mean of agronomic trait from 3 years of data) was performed using the inclusive composite interval mapping (ICIM) procedure with IciMapping version 4.1 (http//www.isbreeding.net), with a scanning step of 1 cM and PIN (probability in stepwise regression) of 0.001 (Li *et al.*, 2007). Permutations (1000 times) were conducted and the LOD threshold was set to 2.5 for both metabolites and agronomic traits (Cui *et al.*, 2017). The confidence interval for each QTL was assigned as a 1.5‐LOD drop of the peak. The additive effect and percentage of phenotypic variance associated with a QTL (contribution) were estimated using the same program. For metabolic QTL (mQTL), the highest PVE was chosen, in which the QTL intervals of the same metabolite overlapped two or three replicates, with one marker extending around the confidence interval. If the phenotypic variance was greater than 15%, it was considered a major QTL (Salvi and Tuberosa, 2005).

The whole genome was divided into 10 cM partitions, and the number of mQTL per partition was counted. Using 1000 permutation tests, each mQTL was randomly assigned to a 10 cM interval, and the number of mQTLs obtained in each interval was counted. The cut‐off number of mQTLs per 10 cM by chance alone was four in mature seeds with *P* < 0.01, respectively. A larger number was regarded as a mQTL hotspot (Gong *et al.*, 2013).

### Vector construction and protein function validation

The genomic DNA was extracted and the candidate gene, *TraesCS2B01G012000.1*, was amplified using the primers PB81 (ATGGACGACGGCCTGGG) + PB37 (TTATTGGCGTTGCACCTTATC) since the candidate only contained a single exon. The sequencing‐confirmed vector was cloned into pGEX‐6p‐1 (Novagen, Madison, WI, USA). Protein induction, cell disruption, and GST protein purification were performed according to the methods reported in Peng *et al. *(2017). The *Strep*II‐tag vector (VB5) was modified from the commercial vector pCXSN‐HA (Taxon ID: 643586) by replacing the HA and ccdB with a *Strep*II coding sequence. Then, the candidate genes were cloned into VB5 using Primer179 (aaattcgtagtggatcccccTTATTGGCGTTGCACCTTATC) and Primer180 (catcctcaatttgaaaaaccaCTGCACATTCCAGAGCAGCA). The resulting constructs were introduced into *Agrobacterium* strain GV3101. Recombinant protein expression and purification were subsequently carried out, as reported by Schroeder *et al. *(2018). The purified protein obtained was then used in an activity assay containing 0.2 m Tris‐HCl (pH 7.5), 10 mm MgCl_2_, 10 mm apigenin, and 0.75 mm UDP‐glucose sampled at 0 and 30 min at 37°C for testing. Different substrate concentrations and time courses were used for the kinetics assay. LC‐MS quantification was used to determine the accumulation of the products. The proteins were then quantified using the Bradford reaction. The values for *K*_M_ and *V*_max_ (to calculated activity levels) were determined using GraphPad PRISM software (GraphPad Software, La Jolla, CA, USA) with the Michaelis–Menten model. The reactions were run in duplicate, and each experiment was repeated twice. Electrophoresis, immunoblotting, and Coomassie Brilliant Blue staining were performed as described in Myrach *et al. *(2017) and Yilamujiang *et al. *(2017).

### Relative expression by qRT‐PCR

The total RNA of 2‐week grain filling seeds was isolated using the plant RNA isolation kit (TIANGEN Biotech, Beijing). Briefly, 1 μg of RNA was treated with DNase and reverse‐transcribed according to the manufacturer’s protocol (EasyScript^®^; TransGen Biotech, Beijing). qRT‐PCR was performed using SYBR Green RT‐PCR Master Mix (Qiagen, Duesseldorf, Germany). Two *Actin* genes were used as an internal control for the quantification of gene expression, amplified by the primers PB140 (ACCCAGATCATGTTCGAGACC) and PB141 (TTCGACCGCTGGCATACAAA) for *Actin‐1D* (*TraesCS1D01G274400*) and PB142 (GCCGTTCTGTCCTTGTATGC) + PB143 (GAGGAAGCGTGTATCCCTCA) for *Actin‐1B* (*TraesCS1B01G283900*). *TraesCS2B01G459900.1* was amplified using the primers PB136 (GACAGGCGCATTCTTGACG) and PB137 (CAGCTCCTCCACGATGAACA). The relative gene expression was calculated as reported by Schroeder *et al. *(2018). Specific primers were designed with the assistance of the Primerserve program (Triticeae Multi‐omics Center).

### Prediction of agronomic traits

The agronomic traits were determined in 2011–2012 (Shijiazhuang), 2012–2013 (Shijiazhuang, Beijing), and 2013–2014 (Shijiazhuang). The materials were planted using a randomized block design with two replications, as previously described in the literature (Zhang *et al.*, 2017; Fan *et al.*, 2019). Briefly, 1260 metabolites (using the average of the three biological replicates of transformed data) were used to predict 17 agronomic traits (using the average of 3 years of replicate data) using the BLUP and LASSO methods (Friedman *et al.*, 2010; Xu *et al.*, 2014). The predictability was measured using a 10‐fold cross‐validation method. The 145 RILs were then randomly divided into 10 groups, 9 of which were used to construct the model. The remaining RILs were predicted. The predictive power (predictability) is defined as the Pearson’s correlation coefficient between the phenotypic observations and the predicted values (Friedman *et al.*, 2010; Xu *et al.*, 2014).

## Data Availability 

All relevant data can be found within the manuscript and its supporting materials.

## Author Contributions

WC and FC conceived the project. WC, TS and AZ designed the experiments. AZ, JJ and TS conducted the experiments. TS, XH and JC performed the main data analysis. WL, XR and DS worked on the field material management. WC and AZ carried out the LC‐MS analyses. AZ, WC and ARF wrote the article.

## Conflicts of Interest

The authors declared that they have no conflicts of interest to this work. We declare that we do not have any commercial or associative interest that represents a conflict of interest in connection with the work submitted.

### Open Research Badges

This article has earned an Open Data Badge for making publicly available the digitally shareable data necessary to reproduce the reported results.

This article has earned an Open Materials Badge for making publicly available the components of the research methodology needed to reproduce the reported procedure and analysis.

## Supporting information


**Figure S1.** Network visualization of 1260 metabolites.
**Figure S2.** The statistical results of mQTL.
**Figure S3.** Distribution of phenotypic variation explained (PVE) about mQTL.
**Figure S4.** Phylogenetic tree of UGT88C13 and UGT706E7.
**Figure S5.** Gene model of the candidate *TraesCS2B01G012000* with the primers used for the amplification.
**Figure S6.** Sequences and alignment of the candidates.
**Figure S7.** Functional annotation of candidate gene *in vitro*.
**Figure S8.** Box plot for *H* ^2^ and CVs about 17 agronomic traits.
**Figure S9.** A common wheat flavone‐related and flavonol‐related metabolic network involving the candidate genes mapped in this study.
**Figure S10.** Genomic data used to predict plant height and number of grains per spike based on two models.

**Table S1.** Scheduled MRM transitions for widely targeted metabolite analysis in mature wheat kernels.
**Table S2.** Metabolic profiling in the kernels of the KN9204/J411 RIL population.
**Table S3.** The statistical results of broad‐sense heritability (*H* ^2^) and coefficient of variation (CV).
**Table S4.** Metabolic quantitative trait loci (mQTLs) results of RIL population.
**Table S5.** Statistical analysis of metabolic quantitative trait loci (mQTLs) on the chromosomes.
**Table S6.** Glucosyltransferase activities and kinetic parameters of UGT88C13 and UGT88C14.
**Table S7.** Pearson’s correlation of 467 annotated metabolite and agronomic traits (*P* < 0.01).
**Table S8.** Agronomic quantitative trait loci (pQTLs) of the RIL population.
**Table S9.** The co‐localization results of pQTLs and mQTLs.
**Table S10.** Metabolites with significant effects on the prediction of plant height and number of grains per spike (*P* < 0.05).
