# Supplementary material for: Metabolomics analysis and metabolite‐agronomic trait associations using kernels of wheat (Triticum aestivum) recombinant inbred lines
Source: Plant J. 2020 Mar 31;103(1):279–92. doi: 10.1111/tpj.14727 (PMC7383920; doi:10.1111/tpj.14727)
Supplement: Supplementary file 1 — Figure S1. Network visualization of 1260 metabolites. Figure S2. The statistical results of mQTL. Figure S3. Distribution of phenotypic variation explained (PVE) about mQTL. Figure S4. Phylogenetic tree of UGT88C13 and UGT706E7. Figure S5. Gene model of the candidate TraesCS2B01G012000 with the primers used for the amplification. Figure S6. Sequences and alignment of the candidates. Figure S7. Functional annotation of candidate gene in vitro. Figure S8. Box plot for H 2 and CVs about 17 agronomic traits. Figure S9. A common wheat flavone‐related and flavonol‐related metabolic network involving the candidate genes mapped in this study. Figure S10. Genomic data used to predict plant height and number of grains per spike based on two models. [file TPJ-103-279-s001.pdf]

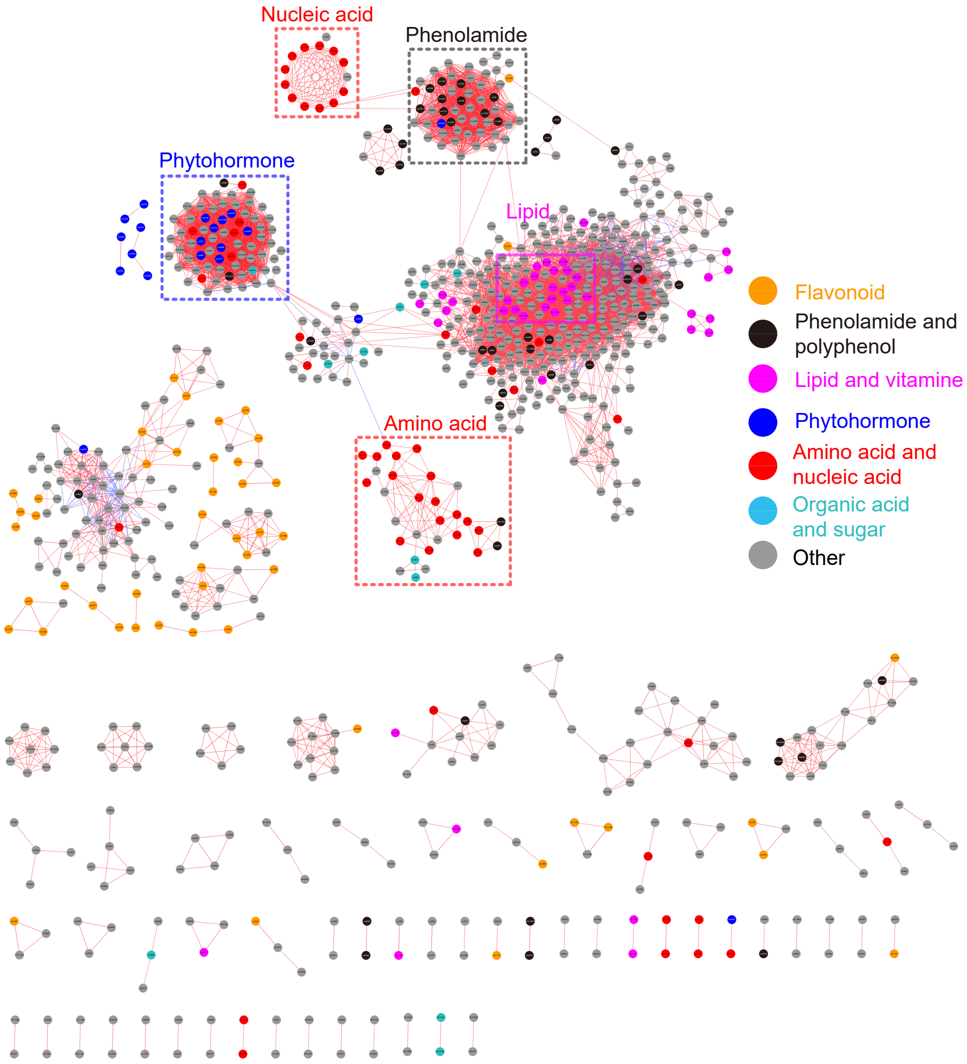

Figure S1 Network visualization of 1260 metabolites.

Metabolites are represented as nodes, and their correlation coefficient values as edges. The absolute values of Pearson's correlation coefficient values above the threshold (r = 0.7) were shown. Red shown positive correlation, and blue shown negative correlation, the depth of colour indicates the correlation level. Metabolites from different chemical groups are marked by distinct colours as shown on right. Fla, flavonoid; PP, phenolamide and polyphenol; LV, lipid and vitamine; Phy, phytohormone; AN, amino acid, nucleic acid and their derivative; OS, organic acid and sugar; Other, unknown classification metabolite.

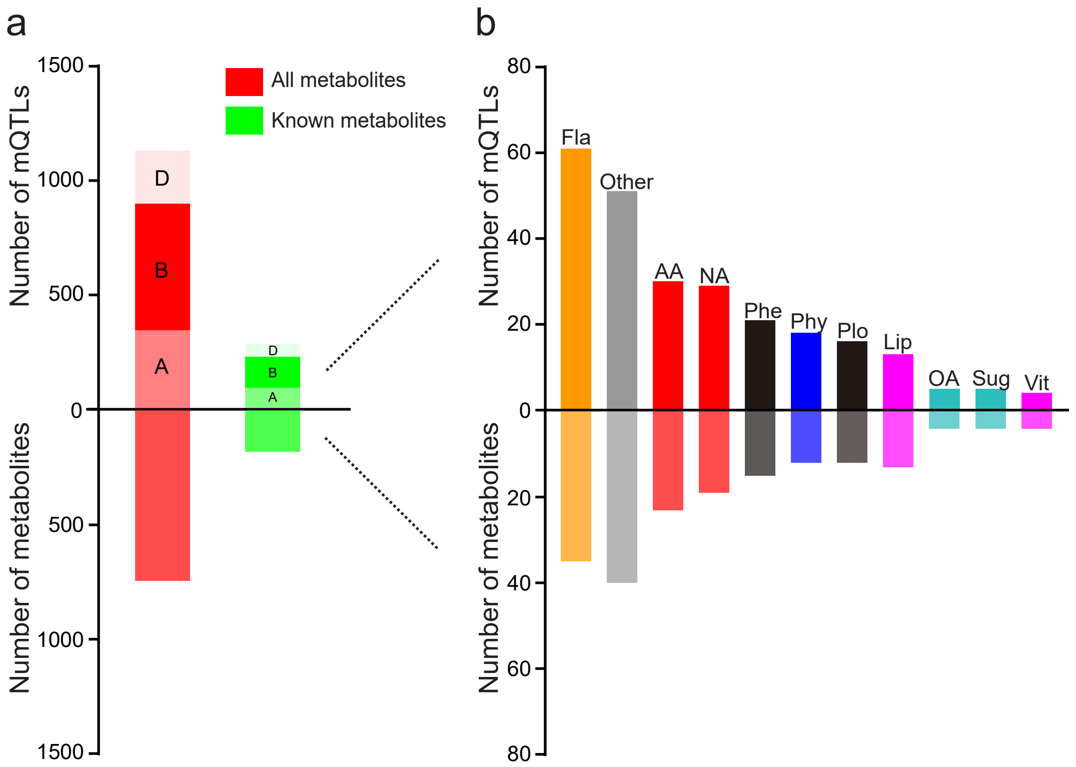

Figure S2 The statistical results of mQTL.

(a) The number and distribution of mQTLs for 746 metabolites (out of the total 1260 metabolites, in red) and 183 metabolites (out of the total 467 known metabolites, in green). A, B, D represent the three sub-genomes of wheat.
(b) The number of mQTLs mapped by different classes of metabolites. Fla, flavonoid; AA, amino acid and its derivative; NA, nucleic acid and its derivative; Phe, phenolamide; Phy, phytohormone; Plo, ployphenol; Lip, lipid; OA, organic acid; Sug, sugar; Vit, vitamine; Other, unknown classification metabolites.

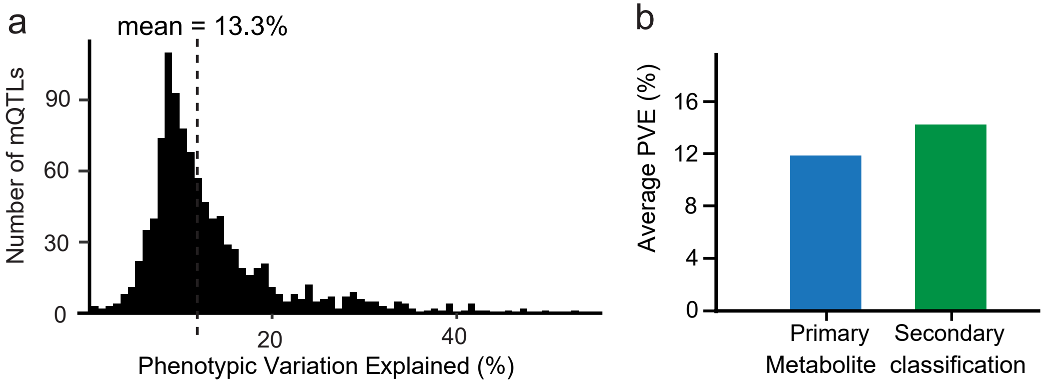

Figure S3 Distribution of Phenotypic Variation Explained (PVE) about mQTL.

(a) The histogram of PVE for 1005 mQTLs in wheat mature seed. The y-axis indicates the number of mQTLs. The image was made using R (http://www.r-project.org/).
(b) Average PVE of primary (in blue) and secondary (in green) metabolites.

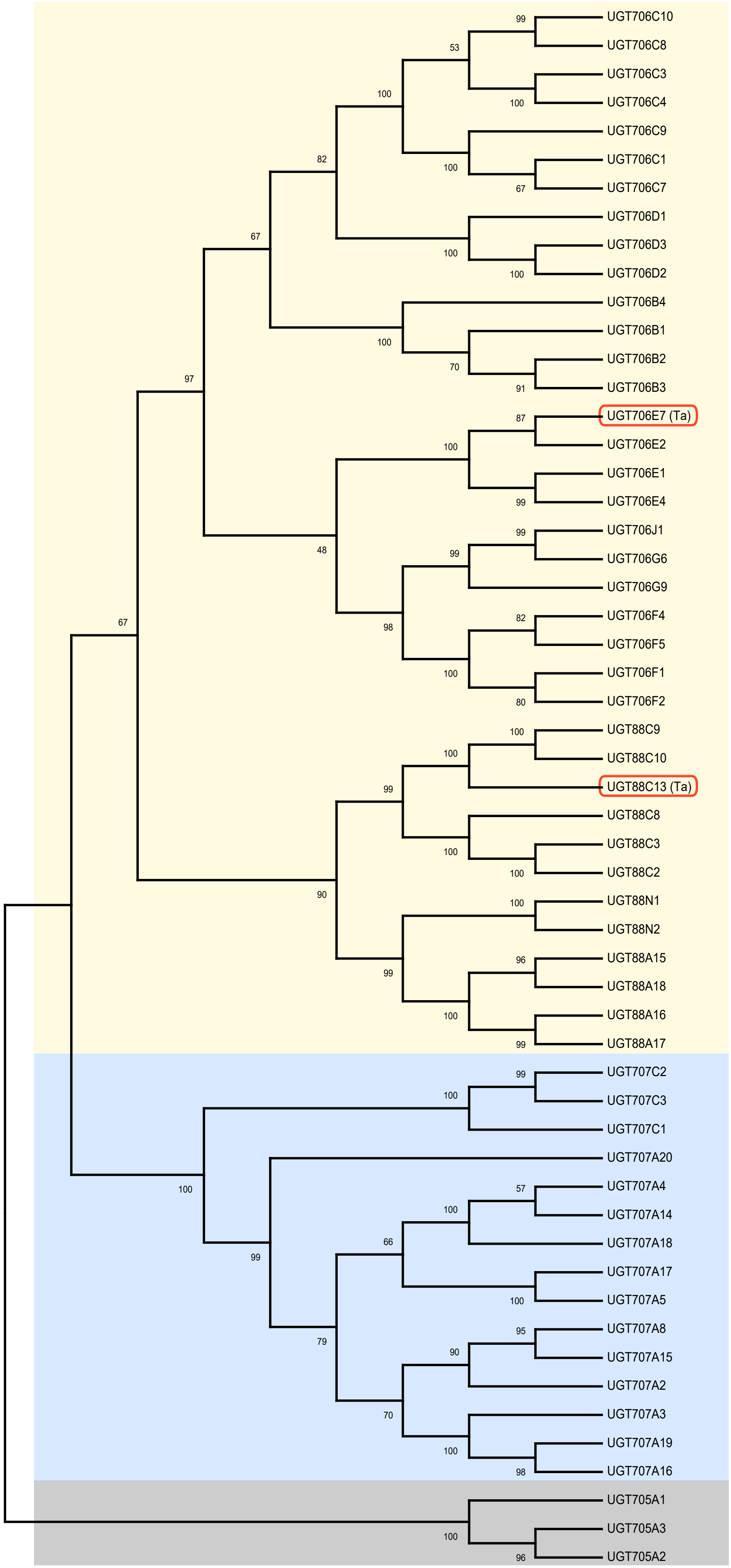

Figure S4 Phylogenetic tree of UGT88C13 and UGT706E7.

Phylogenetic tree of UGT88C13 and UGT706E7corded by *TraesCS2B01G012000* and *TraesCS2B01G459900* constructed with sequences from different UGT members reported previously and the other UGT sequences used in this analysis were the same as described in Peng et al. (2017). The phylogenetic tree has been built by using the neighbor-joining method with the aid of MEGA-X software. Shown is the bootstrap consensus tree (1000 replicas) made by using the model of Jones-Taylor-Thornton. The candidate is labeled in the red box.

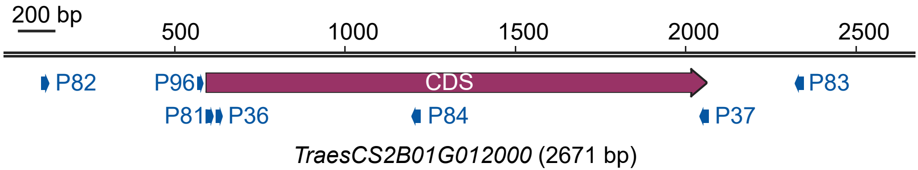

Figure S5 Gene model of the candidate *TraesCS2B01G012000* with the primers used for the amplification.

a

```
>KN9204-2B01G012000-CDS
ATGGACGACGGCCTGGGCACCACCGCCGGGACGCCTCACAAGAAGCGGGTGGTGCTGTACCCGTCGCCGGGGATGGGCCACCTGGT
GTCCATGATCGAGCTCGGCAAGCTCTTCGCGGCGCGGGGCCTGGCCGTCACCATCCTCATCGTGGAGCTGCCCTTCGTCGACACCG
GCGCGCGGGGGCCCTTCCTGGCCGGCGTCACCGCGGCCAACCCGGCCATCTCCTTCCACTGCCTGCCCCGCGTCCGGTTCCCGCCC
CTCGCCTCCCCGCACCCCGAGGCCGTCACCTACGAGGTCGCCCGCCTCTCCAACCCGCACCTCCGCGACTTCCTCCTCGCCGGTGA
TGCCCGTCCGGCCGTCCTCGTCGTCGACTTCTTCTGCAGCGTGGCCCTCGACCTCGCCGCCGAGCTCGGGGTCCCGGGCTACTGCT
TCTTCACGTCCGGCGCCGAGGCCCTGGCGTCCTTCCTGTACCTGCCGGTGCTGCACGAGCAGAGCGCCGCCAGCTTCCGGGAGATG
GGCGAGGAGCTGGTGCGCGTGCCGGGGATCTCGCCGTTCCCGGCCACGCACGCGCTCAAGCCGCTCCAGGACCGCGACGACGCGGC
GTACCGGGGCTTTTTACAGGTTTCACCGGACCTCTGCCGCTCGCAAGGAATCATCATCAACACGTTCCGGTCGCTGGAGCCGCGCG
CCGTCGAGGCGATCGGTGCGGGGCTCTGCACGCCGCCGGGCCTCCCGACGCCGCCGGTGCACTGCATCGGGCCGCTGATAAAGTCG
GCGGAGGTGGGCGTGAAGCGCGGCGGGGAGTGCCTGGCGTGGCTGGACGCGCAGCCGGAGGGCAGTGTAGTGTTCCTCTGCTTCGG
CAGCCTGGGCGTGTTCAGCGCGGCGCAGATCAGGGAGATCGGCGTCGGGCTGGAGGCGAGCGGCGTGAGGTTTCTATGGGTGGTCC
GCAGCCCGCCGAACGAGGACCCGGCCAAGAGGTTCGAGGAGCCGCCGGAGCCGGACCTCGGCGCGCTGCTCCCGGAGGGCTTCCTG
GAGCGGACCGGAGAAAGAGGGCTGGTGGTGAAGACGTGGGCGCCGCAGCGGGACTTGCTGGCGCACGGCGCGGTGGGCGGGTTCGT
GACGCACTGCGGCTGGAACTCGGTGCTGGAGGCGGTGATGGCCGGCGTGCCGATGCTGGCGTGGCCGCTGTACGCGGAGCAGCGGC
TGAACCGGGTGTTCCTGGAGAAGGAGCTGGGGCTGGCCGCGGCGGTGGAGGGGTACGACCAAGAGGGTGAGGTGGTGGAGGCCGGC
GAGGTGGAGAAGAAGGTGCGGTGGCTGATGGAGTCCGACGGCGGGAGCGTGCTCCGGGAGCGCACGCTGGGCACCATGAGGAGGGC
CAAGGAGGCGCTGGCCGCGGGCGGGGAGTCGGACGTGACGCTCACCAAGCTGGTGGAGGGCTGGATGGGAGACGATAAGGTGCAAC
GCCAATAA
```

b

```
UGT88C13    1 MDDGLGTTAGTPHKKRVVLYPSPGMGHLVSMIELGKLFAARGLAVTILIVELPFVDTGAR
UGT88C14    1 MDDGLGTTAGTPHKKRVVLYPSPGMGHLVSMIELGKLFAARGLAVTILIVELPFVDTGAR

UGT88C13   61 GPFLAGVTAANPAISFHCLPRVRFPPLASSHPEAVTYEVARLSNPHLRDFLLAGDARPAV
UGT88C14   61 GPFLAGVTAANPAISFHCLPRVRFPPLASPHPEAVTYEVARLSNPHLRDFLLAGDARPAV

UGT88C13  121 LVVDFFCSVALDLAAELGVPGYCFFTSGAEALASFLYLPVLHEQSAASFREMGEELVRVP
UGT88C14  121 LVVDFFCSVALDLAAELGVPGYCFFTSGAEALASFLYLPVLHEQSAASFREMGEELVRVP

UGT88C13  181 GISPFPATHALKPLQDRDDAAYRGFLQVSPDLCRSQGIIINTFRSLEPRAVEAIGAGLCT
UGT88C14  181 GISPFPATHALKPLQDRDDAAYRGFLQVSPDLCRSQGIIINTFRSLEPRAVEAIGAGLCT

UGT88C13  241 PPGLPTPPVHCIGPLIKSAEVGVKRGGECLAWLDAQPEGSVVFLCFGSLGVFSAAQIREI
UGT88C14  241 PPGLPTPPVHCIGPLIKSAEVGVKRGGECLAWLDAQPEGSVVFLCFGSLGVFSAAQIREI

UGT88C13  301 GVGLEASGVRFLWVVRSPPNEDPAKRFEEPPEPDLGALLPEGFLERTGERGLVVKTWAPQ
UGT88C14  301 GVGLEASGVRFLWVVRSPPNEDPAKRFEEPPEPDLGALLPEGFLERTGERGLVVKTWAPQ

UGT88C13  361 RDVLAHGAVGGFVTHCGWNSVLEAVMAGVPMLAWPLYAEQRLNRVFLEKELGLAAAVEGY
UGT88C14  361 RDLLAHGAVGGFVTHCGWNSVLEAVMAGVPMLAWPLYAEQRLNRVFLEKELGLAAAVEGY

UGT88C13  421 DQEGEVVEAGEVEKKVRWLMESDGGSVLRERTLGAMRRAKEALAAGGESDVTLTKLVEGW
UGT88C14  421 DQEGEVVEAGEVEKKVRWLMESDGGSVLRERTLGTMRRAKEALAAGGESDVTLTKLVEGW

UGT88C13  481 MGDDKVQRQ
UGT88C14  481 MGDDKVQRQ
```

Figure S6 Sequences and alignment of the candidates.

(a) Nucleotide sequence of CDS of the candidate gene *2B01G012000* amplified from KN9204.
(b) Alignment of the corresponding candidate proteins UGT88C13 (corded by *TraesCS2B01G012000*) and UGT88C14 (coded by the sequence in S4a).

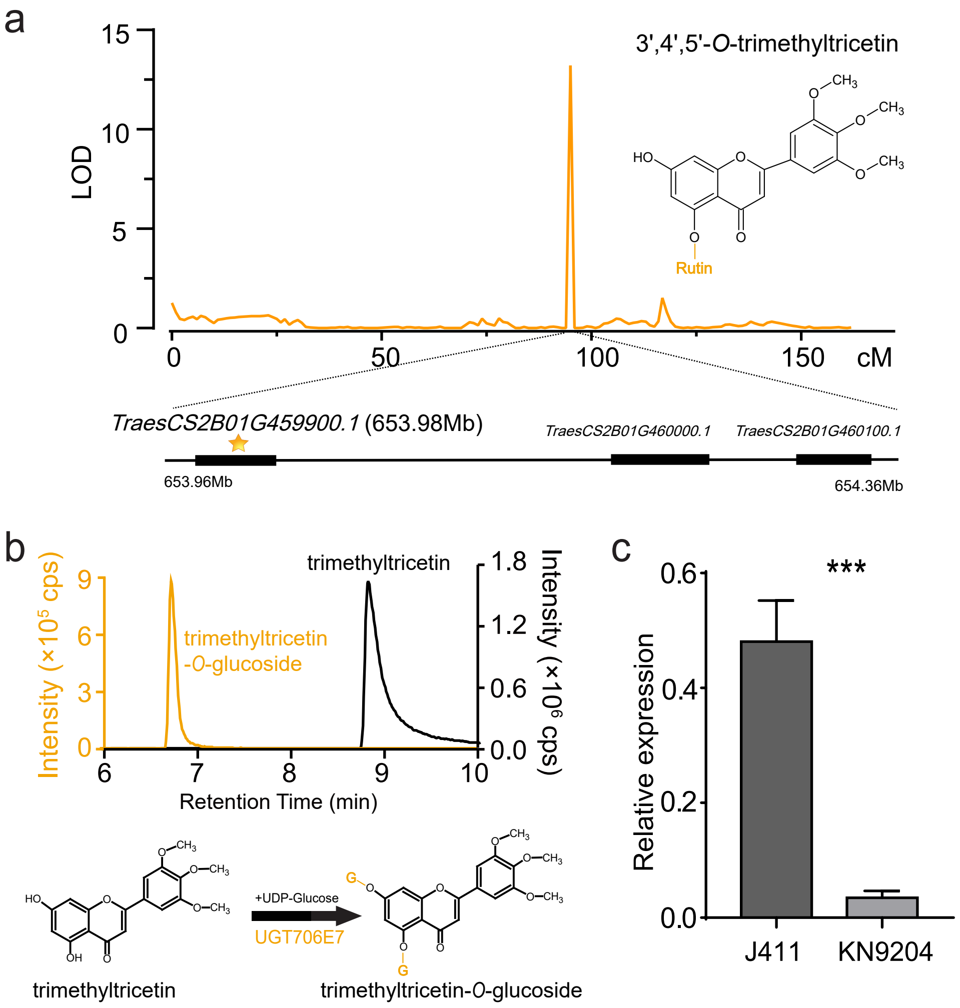

Figure S7 Fuctional annotation of candidate gene *in vitro*.

(a) LOD curves of QTL mapping of the 3’,4’,5’-Tricetin *O*-rutinoside accumulation on chromosome 2B and the gene model of *TraesCS2B01G459900.1* with a star indicating its position.
(b) Chromatograms of the *in vitro* assay by the coded protein UGT706E7.
(c) Relative expression of the targeted gene in two parental lines J411 and KN9204 (*P* < 0.001; *t* test). Quantified from three biological replicates and two technical replicate error bars indicate standard deviation.

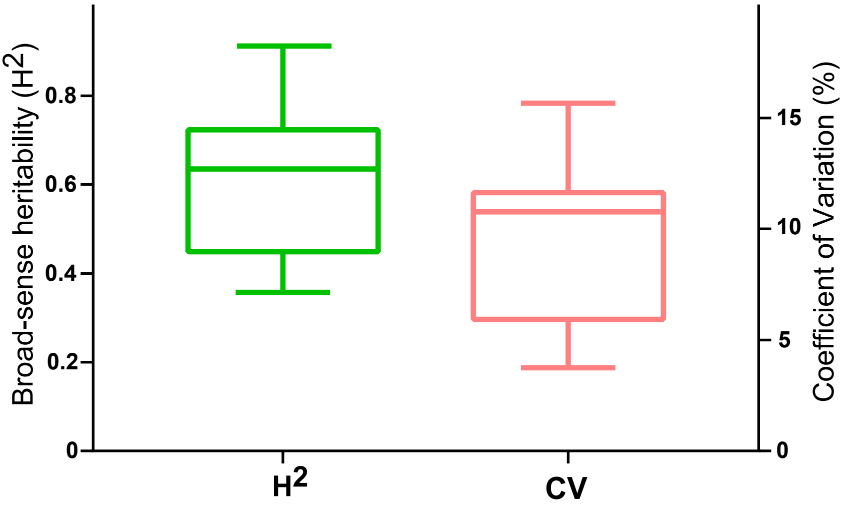

Figure S8 Box plot for H^2 and CVs about 17 agronomic traits.

The broad-sense heritability (H^2) and coefficent of variation (CV) for agronomic traits are shown in green and pink, respectively. The image was made using GraphPad Software (https://www.graphpad.com).

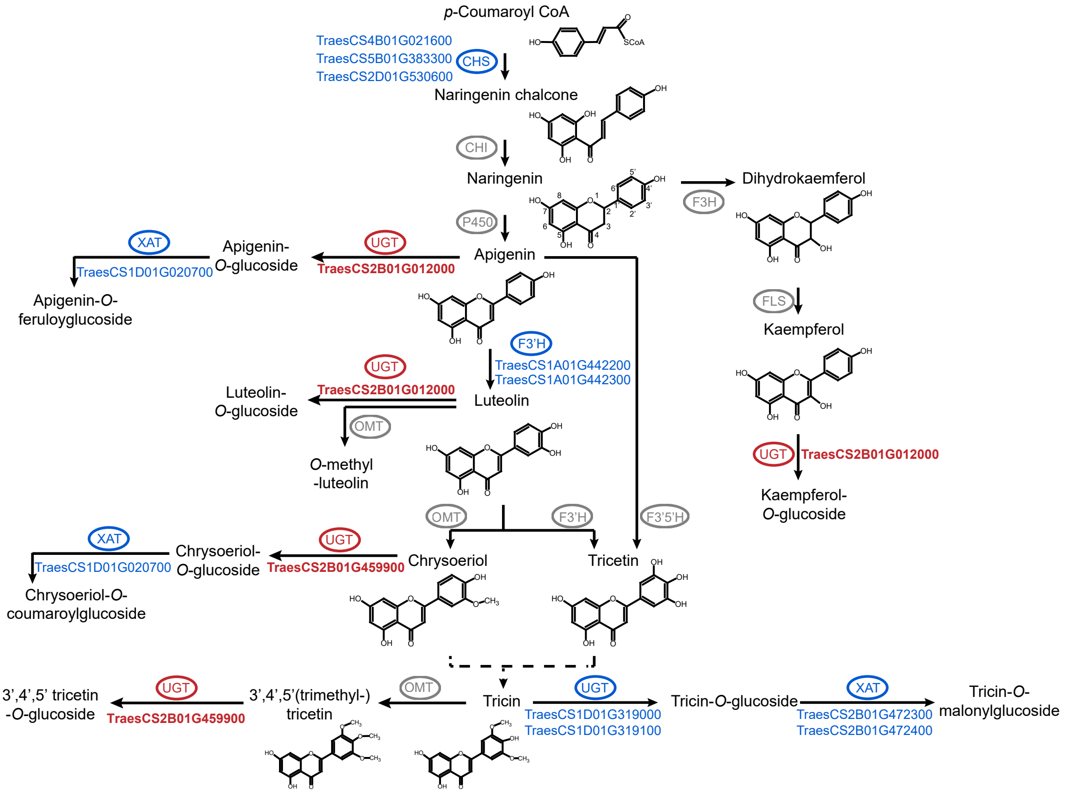

Figure S9 A common wheat flavone- and flavonol-related metabolic network involving the candidate genes mapped in this study.

Grey coloured enzyme abbreviations indicate un-characterized proteins in common wheat; blue ones are in the candidate list of this study (with more details in supplemental table S4), and the candidate genes in red were identified in vitro. CHS: chalcone synthase; UGT: UDP-glucuronosyltransferase; F3'H: flavonoid 3'-hydroxylase; OMT: O-methyltransferase; XAT: Acyltransferase.

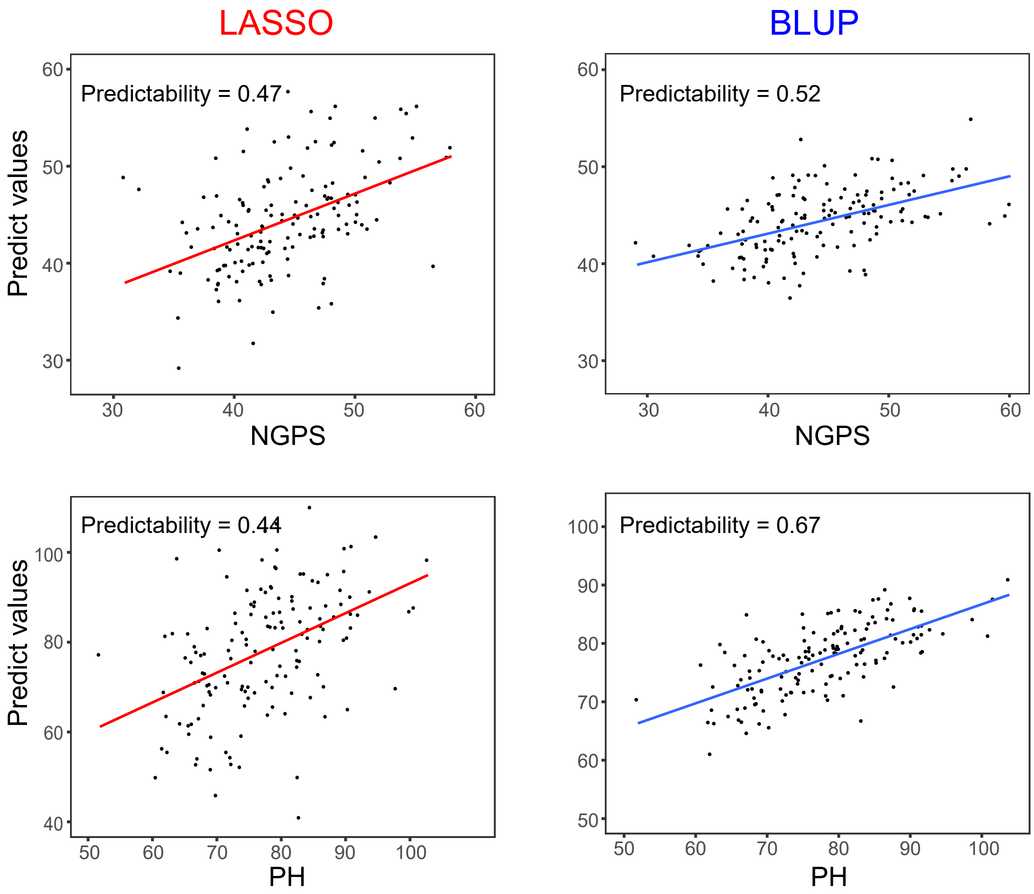

Figure S10 Genomic data used to predict plant height and number of grains per spike based on two models.

The BLUP and LASSO models were used to predict the plant height and number of grains per spike, respectively. Right: BULP prediction result. Left: LASSO prediction result. The x-axis indicates the predictive value of agronomic traits and the y-axis indicates phenotypic observations. The image was made using R (http://www.r-project.org/).
