# Supplementary material for: Metabolomics analysis and metabolite‐agronomic trait associations using kernels of wheat (Triticum aestivum) recombinant inbred lines
Source: Plant J. 2020 Mar 31;103(1):279–92. doi: 10.1111/tpj.14727 (PMC7383920; doi:10.1111/tpj.14727)
Supplement: Supplementary file 3 [file TPJ-103-279-s003.docx]

**Figure S1. Network visualization of 1260 metabolites.**

Metabolites are represented as nodes, and their correlation coefficient values as edges. The absolute values of Pearson’s correlation coefficient values above the threshold (*r* = 0.7) were shown. Red shown positive correlation, and blue shown negative correlation, the depth of colour indicates the correlation level. Metabolites from different chemical groups are marked by distinct colours as shown on right. Fla, flavonoid; PP, phenolamide and polyphenol; LV, lipid and vitamine; Phy, phytohormone; AN, amino acid, nucleic acid and their derivative; OS, organic acid and sugar; Other, unknown classification metabolite.

**Figure S2. The statistical results of mQTL.**

(a) The number and distribution of mQTLs for 746 metabolites (out of the total 1260 metabolites, in red) and 183 metabolites (out of the total 467 known metabolites, in green). A, B, D represent the three sub-genomes of wheat. (b) The number of mQTLs mapped by different classes of metabolites. Fla, flavonoid; AA, amino acid and its derivative; NA, nucleic acid and its derivative; Phe, phenolamide; Phy, phytohormone; Plo, ployphenol; Lip, lipid; OA, organic acid; Sug, sugar; Vit, vitamine; Other, unknown classification metabolites.

**Figure S3. Distribution of Phenotypic Variation Explained (PVE) about mQTL.**

(a) The histogram of PVE for 1005 mQTLs in wheat mature seed. The y-axis indicates the number of mQTLs. The image was made using R (http://www.r-project.org/). (b) Average PVE of primary (in blue) and secondary (in green) metabolites.

**Figure S4. Phylogenetic tree of UGT88C13 and UGT706E7.**

Phylogenetic tree of UGT88C13 and UGT706E7corded by *TraesCS2B01G012000* and *TraesCS2B01G459900* constructed with sequences from different UGT members reported previously and the other UGT sequences used in this analysis were the same as described in Peng et al. (2017). The phylogenetic tree has been built by using the neighbor-joining method with the aid of MEGA-X software. Shown is the bootstrap consensus tree (1000 replicas) made by using the model of Jones-Taylor-Thornton. The candidate is labeled in the red box.

**Figure S5. Gene model of the candidate *TraesCS2B01G012000* with the primers used for the amplification.**

**Figure S6. Sequences and alignment of the candidates.**

(a) Nucleotide sequence of CDS of the candidate gene *2B01G012000* amplified from KN9204. (b) Alignment of the corresponding candidate proteins UGT88C13 (corded by *TraesCS2B01G012000*) and UGT88C14 (coded by the sequence in S4a).

**Figure S7. Functional annotation of candidate gene *in* *vitro*.**

(a) LOD curves of QTL mapping of the 3’,4’,5’-Tricetin *O*-rutinoside accumulation on chromosome 2B and the gene model of *TraesCS2B01G459900.1* with a star indicating its position. (b) Chromatograms of the *in vitro* assay by the coded protein UGT706E7. (c) Relative expression of the targeted gene in two parental lines J411 and KN9204 (*P* < 0.001; *t* test). Quantified from three biological replicates and two

technical replicate error bars indicate standard deviation.

**Figure S8. Box plot for *H^2^* and CVs about 17 agronomic traits.**

The broad-sense heritability (*H^2^* ) and coefficent of variation (CV) for agronomic traits are shown in green and pink, respectively. The image was made using GraphPad Software (https://www.graphpad.com).

**Figure S9. A common wheat flavone- and flavonol-related metabolic network involving the candidate genes mapped in this study.**

Grey coloured enzyme abbreviations indicate un-characterized proteins in common wheat; blue ones are in the candidate list of this study (with more details in supplemental table S4), and the candidate genes in red were identified in vitro. CHS: chalcone synthase; UGT: UDP-glucuronosyltransferase; F3’H: flavonoid 3'-hydroxylase; OMT: O-methyltransferase; XAT: Acyltransferase.

**Figure S10. Genomic data used to predict plant height and number of grains per spike based on two models.**

The BLUP and LASSO models were used to predict the plant height and number of grains per spike, respectively. Right: BULP prediction result. Left: LASSO prediction result. The x-axis indicates the predictive value of agronomic traits and the y-axis indicates phenotypic observations. The image was made using R (http://www.r-project.org/).

**Table S1.** Scheduled MRM transitions for widely targeted metabolite analysis in mature wheat kernels.

**Table S2.** Metabolic profiling in the kernels of the KN9204/J411 RIL population.

**Table S3.** The statistical results of broad-sense heritability (*H^2^*) and coefficient of variation (CV).

**Table S4.** Metabolic quantitative trait loci (mQTLs) results of RIL population.

**Table S5.** Statistical analysis of metabolic quantitative trait loci (mQTLs) on the chromosomes.

**Table S6.** Glucosyltransferase activities and kinetic parameters of UGT88C13 and UGT88C14.

**Table S7.** Pearson’s correlation of 467 annotated metabolite and agronomic traits (*P* < 0.01).

**Table S8.** Agronomic quantitative trait loci (pQTLs) of the RIL population.

**Table S9.** The co-localization results of pQTLs and mQTLs.

**Table S10.** Metabolites with significant effects on the prediction of plant height and number of grains per spike (*P* < 0.05).
